# Aberrant DNA Methylation of *SEPT9* and *SDC2* in Stool Specimens as an Integrated Biomarker for Colorectal Cancer Early Detection

**DOI:** 10.3389/fgene.2020.00643

**Published:** 2020-06-18

**Authors:** Guodong Zhao, Xiaoyu Liu, Yi Liu, Hui Li, Yong Ma, Shiming Li, Yun Zhu, Jin Miao, Shangmin Xiong, Sujuan Fei, Minxue Zheng

**Affiliations:** ^1^Zhejiang University Kunshan Biotechnology Laboratory, Zhejiang University Kunshan Innovation Institute, Kunshan, China; ^2^State Key Laboratory of Bioelectronics, School of Biological Science and Medical Engineering, Southeast University, Nanjing, China; ^3^Suzhou VersaBio Technologies Co., Ltd., Kunshan, China; ^4^Department of Gastroenterology, Affiliated Hospital of Xuzhou Medical University, Xuzhou, China; ^5^Institute of Digestive Diseases, Xuzhou Medical University, Xuzhou, China; ^6^Suzhou Institute of Biomedical Engineering and Technology, Chinese Academy of Sciences, Suzhou, China

**Keywords:** colorectal cancer, stool, m*SEPT9*, m*SDC2*, early detection

## Abstract

Colorectal cancer (CRC) has become the second leading cause of new cancer cases and the fifth of cancer deaths in China, and early detection is the most effective way to reduce the incidence and mortality of CRC. A number of methylated DNA biomarkers have been found to associate with CRC and precancerous lesions in stool samples, indicating stool methylated DNA biomarkers are potential tools for CRC early detection. In this study, approximately 5 g of stool specimen was collected from 230 subjects (124 in the training set and 106 in the validation set). Stool DNA was extracted and bisulfite-converted, followed by ColoDefense test, a multiplex qPCR assay, that simultaneously detects methylated *SEPT9* (m*SEPT9)* and methylated *SDC2* (m*SDC2*). Youden index was employed to determine the cut-off value of ColoDefense test for stool specimens. In the training set, the optimized cut-off value of stool ColoDefense test was: m*SEPT9* analyzed with 3/3 algorithm and mean m*SEPT9* Ct values of <38, or m*SDC2* with 2/3 algorithm. Stool ColoDefense test achieved Youden indexes of 79.9 and 57.4% in detecting CRC and advanced adenomas (AA), respectively. Its sensitivities in the training set for AA and CRC were 66.7% (95% CI: 24.1–94.0%) and 89.1% (95% CI: 77.1–95.5%) with a 90.8% (95% CI: 80.3–96.2%) specificity, and AUC was 0.956 (95% CI: 0.924–0.988). In the validation set, its sensitivities for AA and CRC were 66.7% (95% CI: 24.1–94.0%) and 92.3% (95% CI: 78.0–98.0%) with a 93.2% (95% CI: 82.7–97.8%) specificity, and AUC was 0.977 (95% CI: 0.952–1.000). Positive detection rate of stool ColoDefense test has been found to be independent of age, gender, tumor location, and tumor size. In conclusion, stool ColoDefense test demonstrated high sensitivities and specificity for the detection of AA and CRC. Therefore, it has the potential to become a low-cost, convenient, and highly effective tool for CRC early detection.

## Introduction

Approximately 2 million new colorectal cancer (CRC) cases and over 881,000 deaths were estimated to occur in 2018 worldwide, accounting for about 1 in 10 new cancer cases and deaths. Overall, CRC ranked the third in terms of incidence but the second in terms of mortality all over the world (Bray et al., 2018). While in China, Over 517,000 new CRC cases and more than 245,000 CRC deaths were estimated for the same year, ranking the second in terms of incidence and the fifth in terms of mortality by the same study (Ferlay et al., 2018). And CRC has been reported to be more common in male aged 60 years in China (Chen et al., 2018).

Early detection is one of the most effective ways to reduce the incidence and mortality of CRC, therefore CRC early screening programs are organized in several countries (Sano et al., 2016). In China, a two-step screening strategy has been recommended for population based CRC screening, the guaiac-based fecal occult blood test (gFOBT) and a quantitative high-risk factor questionnaire as the primary screening, with a full colonoscopy for follow-up (Sano et al., 2016). However, the benefit of gFOBT is limited due to its low sensitivity of 33.3–57.1% (Tinmouth et al., 2015). Colonoscopy, as the gold standard for CRC diagnosis, has been widely used in early CRC screening programs in many countries (Mohammadi et al., 2017). For the past 5 years, CRC incidence has continued to decline by approximately 3% every year in the US due to the increased acceptance of colonoscopy, which allows removal of precancerous lesions (Siegel et al., 2012, 2019). Colonoscopy among US adults elder than 50 years tripled from 21% in 2000 to 60% in 2015 (Siegel et al., 2019). In contrast, a recent population-based CRC screening program in China revealed a relatively low participation rate due to its invasiveness, bothersome bowel preparation and difficult-to-avoid complications (Chen H. et al., 2019).

DNA methylation is known to be abnormal in many cancers (Klutstein et al., 2016). A number of methylated DNA biomarkers have been found to associate with CRC and precancerous lesions in stool or plasma samples, including *SEPT9* (Catherine et al., 2008; Lamb and Dhillon, 2017), *SDC2* (Barták et al., 2017; Han et al., 2019), *SFRP2* (Barták et al., 2017; Li et al., 2019), and *TFPI2* (Glöckner et al., 2009), some of which have been developed into commercial kits for CRC early detection (Potter et al., 2014; Lamb and Dhillon, 2017; Li et al., 2019; Zhao et al., 2019). For example, plasma methylated *SEPT9* (m*SEPT9*) test (Epi proColon 2.0 assay) was approved for CRC early detection by FDA in 2016 (Lamb and Dhillon, 2017). Meanwhile, Cologuard, a stool DNA test approved by FDA, also includes two methylated DNA biomarkers (Imperiale et al., 2014), and it has been recommended by the most recent CRC screening guideline in the US (Wolf et al., 2018). We previously demonstrated a multiplex methylated DNA test in plasma, ColoDefense test, with high sensitivity and specificity for CRC early detection. It detects m*SEPT9*, methylated *SDC2* (m*SDC2*), and an internal control (*ACTB*) simultaneously in a single reaction (Chen Y. et al., 2019; Zhao et al., 2019). In previous studies, plasma m*SEPT9* test showed low sensitivities in detecting early stage CRC and advanced adenomas (AA) (Potter et al., 2014; Timothy et al., 2014). Instead, we have demonstrated that the detection rates of plasma ColoDefense test for AA and early stage CRC were significantly improved by the combination of two biomarkers, m*SEPT9* and m*SDC2*, with high specificity (Zhao et al., 2019). However, plasma ColoDefense test may be a better choice for hospitals, because blood draw is convenient to perform for medical personnel, but requires an appointment in advance which takes more time. While stool samples are convenient to collect at home, it can provide more privacy for those who are concerned, or too busy, or too afraid of a cross-infection (like the Covid-19) to go to the hospital. Therefore, combination of plasma and stool tests can facilitate broader population to participate in the early diagnosis of colorectal cancer. In this study, we evaluated the feasibility of m*SEPT9* and m*SDC2* in stool specimens as an integrated biomarker for early CRC detection, and optimized its cut-off value.

## Materials and Methods

### Sample Collection

**FIGURE 1:**
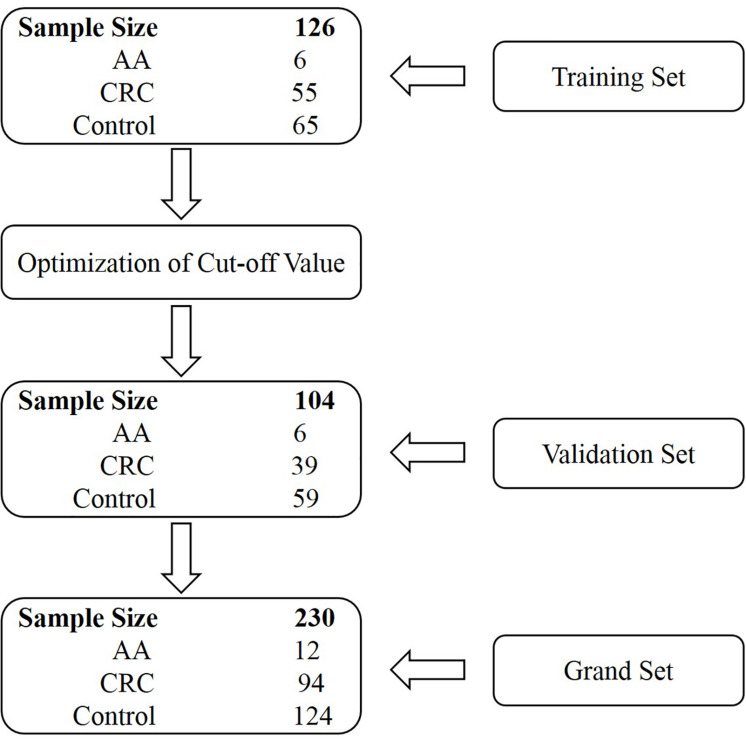
Schematic representation of the study design.

Stool specimens were collected from 230 subjects who underwent colonoscopy at the Affiliated Hospital of Xuzhou Medical University. Participants were prospectively enrolled in two independent cohorts, the training and validation sets. The training set comprised subjects enrolled from July 1, 2018 until February 1, 2019, and the validation set consisted of subjects enrolled from March 1, 2019 until December 1, 2019 (Figure 1). The training set included 55 CRC patients, 6 AA (adenomas measuring ≥1 cm in the greatest dimension or with high-grade dysplasia or with ≥25% villous histologic features) patients and 65 control subjects (colonoscopy negative subjects). The validation set included 39 CRC patients, 6 AA patients, and 59 control subjects (Figure 1 and Supplementary Tables 1, 2). Some CRC and AA patients were tested positive for gFOBT or colonoscopy before being transferred to the Affiliated Hospital of Xuzhou Medical University. All stool samples were collected prior to purgative bowel preparation or colonoscopy. Whole stools were collected in single-use disposable buckets mounted on toilet seats, and approximately 5 g of each stool specimen was transferred into a 50 mL tube containing 25 mL of preservative buffer (Suzhou VersaBio Technologies Co., Ltd., Kunshan, China) to stabilize human genomic DNA in the stool. All stool samples were stored at room temperature for no more than 7 days or −80°C for longer-term storage before usage.

This study was approved by the Institutional Review Board of the Affiliated Hospital of Xuzhou Medical University (Ethics Committee reference number: XYFY2018-KL081), and the informed consent was obtained for all participating patients and control subjects.

### DNA Extraction, Bisulfite Treatment, and Quantitative Real-Time PCR

All stool samples were thawed for about 30 min at 15–30°C and subsequently homogenized for 1 min with a shaking device. After homogenization, each stool sample was centrifuged for 20 min at 10,000 g. One hundred and fifty microliters supernatants were removed for human genomic DNA extraction with a stool DNA extraction kit (Suzhou VersaBio Technologies Co., Ltd.). Briefly, each supernatant was added with 500 μL fresh preservative buffer and centrifuged at 20,000 g for 3 min. The resulting supernatant was then transferred to a new tube, and 600 μL lysis buffer and 20 μL proteinase K solution were added to each sample followed by incubation at 70°C for 10 min. Next, each sample was added with 600 μL absolute ethanol and then loaded onto a spin column. After two washing steps, the column was dried and DNA was eluted with 100 μL elution buffer. Bisulfite conversion of purified DNA and purification of the converted products were performed with a fast bisulfite conversion kit (Suzhou VersaBio Technologies Co., Ltd.). Briefly, 150 μL conversion buffer and 25 μL protection buffer were added to 100 μL purified DNA solution, and followed by incubation at 80°C for 45 min. Next, 1 mL wash buffer A was added to each sample and loaded onto a spin column. After two washing steps, the column was air dried and DNA was eluted with 100 μL elution buffer.

Purified and converted DNA from the above steps was tested by ColoDefense test developed by Suzhou VersaBio Technologies Co., Ltd. Three PCR replicates were performed for each sample. The total volume of the ColoDefense test was 30 μL including 15 μL template and 15 μL PCR master mix. qPCR was analyzed on LC480-II thermal cycler (Roche Diagnostics, Basel, Switzerland) using the following cycling conditions: activation at 95°C for 30 min, 50 cycles of 95°C for 10 s, 56°C for 30 s, and final cooling to 40°C for 30 s (Zhao et al., 2019).

### Data Analysis

*ACTB* was used as the internal control gene for valid sample collection and processing to avoid false negatives. The result for a stool sample was considered “invalid” if *ACTB* Ct was greater than 41.0, and m*SEPT9* and m*SDC2* were considered “detected” if their Ct values were less than 45.0 and 50.0, respectively. As ColoDefense test is a multiplex qPCR reaction run in triplicates and therefore returns with several possible results depending on different scoring algorithms (1/3, 2/3, or 3/3 for each target). According to this principle, the results of ColoDefense test were analyzed with different algorithms to determine the optimal algorithm (Tables 2, 3). Youden index (sensitivity + specificity - 1) was employed to determine cutoff values, where specificity equals to 1 minus positive detection rate of control, and sensitivity equals to positive detection rate of CRC or AA. Therefore, Youden index of CRC or AA equals to positive detection rate of CRC or AA minus positive detection rate of control.

Statistical analysis was performed using IBM SPSS for Windows, Version 22.0, and *t*-test was used for comparison between two testing subjects at the significance level of *p* <0.05. Receiver operating characteristic (ROC) curves were plotted using the mean Ct values from CRC and control subjects. Because m*SEPT9* and m*SDC2* were not detected from most control subjects by the qPCR reaction, we set the corresponding Ct values to 50.0 (the maximal number of PCR cycles) for such samples to plot the curves (Wu et al., 2016).

## Results

Two hundred and thirty participants in total were originally enrolled in our study, whose baseline characteristics are shown in Table 1. The training set included 126 subjects, among which 55 were CRC patients including one stage 0, 11 stage I, 15 stage II, 18 stage III, four stage IV patients and six patients of unknown stage. For the validation set, there were 39 CRC patients including nine stage I, eight stage II, 16 stage III, three stage IV patients and three patients of unknown stage (Supplementary Tables 1, 2).

**TABLE 1:** Subjects characteristics.

	**Training set**		**Validation set**	
**Age (years)**	**Min-Max**	**Mean**	**Min-Max**	**Mean**
AA	47–75	62.2	46–67	56.2
CRC	35–86	60.0	27–83	59.0
Control	24–69	45.2	24–83	47.9

**Gender [N (%)]**	**Male**	**Female**	**Male**	**Female**

AA	5 (83.3)	1 (16.7)	3 (50.0)	3 (50.0)
CRC	32 (58.2)	23 (41.8)	21 (53.8)	18 (46.2)
Control	31 (47.7)	34 (52.3)	31 (52.5)	28 (47.4)

**TABLE 2:** Positive detection rates of m*SEPT9* and m*SDC2* with 1/3, 2/3, and 3/3 algorithms for AA, CRC, and control groups.

**Algorithm**	**Group**	**Subject number (n)**	**Positive detection rate (95% CI:%)**	
			**m*SEPT9***	**m*SDC2***
1/3	AA	6	100.0 (51.7–100.0)	50.0 (13.9–86.1)
	CRC	55	98.2 (89.0–99.9)	90.9 (79.3–96.6)
	Control	65	53.8 (41.1–66.1)	26.2 (16.4–38.8)
2/3	AA	6	100.0 (51.7–100.0)	33.3 (6.0–75.9)
	CRC	55	94.5 (83.9–98.6)	83.6 (70.7–91.8)
	Control	65	41.5 (29.7–54.4)	6.2 (2.0–15.8)
3/3	AA	6	83.3 (36.5–99.1)	33.3 (6.0–75.9)
	CRC	55	85.5 (72.8–93.1)	78.2 (64.6–87.8)
	Control	65	23.1 (13.9–35.5)	0.0 (0.0–7.0)

The cut-off value of ColoDefense test for plasma specimens is the detection of m*SEPT9* in 1 or m*SDC2* in 2 out of 3 replicate PCR reactions (Zhao et al., 2019). However, m*SEPT9* showed low specificity with 1/3 algorithm for stool specimens (Table 2). Therefore, we used a two-step approach to optimize the cut-off value of ColoDefense test for stool specimens. First, we analyzed the performance of m*SEPT9* and m*SDC2* with different algorithms (Table 2). Similar to the results for plasma specimens, m*SDC2* showed relatively high sensitivity (83.6%) and specificity (93.8%) in detecting CRC with 2/3 algorithm. However, with 1/3 algorithm, m*SDC2* resulted in higher sensitivity in detecting both AA and CRC but a lower specificity. In contrast, with 3/3 algorithm, m*SDC2* showed a 100% specificity and a 78.2% sensitivity, even better than its performance for plasma specimens. As for m*SEPT9*, it showed very high sensitivities and low specificities in detecting both AA and CRC with 1/3, 2/3 and 3/3 algorithms.

While the combination of m*SEPT9* and m*SDC2* could achieve higher sensitivity than m*SEPT9* or m*SDC2* alone, it would decrease specificity. Therefore, in the second step, we gradually tightened the Ct requirement for m*SEPT9*, and the resulting m*SEPT9* readouts were combined with m*SDC2* in 2/3 and 3/3 algorithms to choose the optimal cut-off value. As shown in Table 3, m*SEPT9* with 3/3 algorithm and a mean m*SEPT9* Ct value of less than 38 combined with m*SDC2* with 2/3 algorithm achieved the relatively high Youden index for detecting CRC (79.9%) and AA (57.4%), showed a balance of sensitivities and specificity. Therefore, all subsequent data were analyzed with these criteria.

**TABLE 3:** Positive detection rates of ColoDefense test with different algorithm combinations for m*SEPT9* and m*SDC2* in detecting AA, CRC, and control.

**Algorithm**		**Group**	**Subject number (n)**	**Positive detection rate (95% CI:%)**	**Youden index (%)**
**m*SEPT9***	**m*SDC2***				
3/3	2/3	AA	6	83.3 (36.5–99.1)	54.1
		CRC	55	94.5 (83.9–98.6)	65.3
		Control	65	29.2 (18.9–42.0)	N/A
3/3	3/3	AA	6	66.7 (24.1–94.0)	42.1
		CRC	55	90.9 (3.4–20.7)	66.3
		Control	65	24.6 (15.1–37.1)	N/A
3/3 and mean Ct <40	2/3	AA	6	83.3 (36.5–99.1)	60.3
		CRC	55	90.9 (3.4–20.7)	67.8
		Control	65	23.1 (13.9–35.5)	N/A
3/3 and mean Ct <40	3/3	AA	6	66.7 (24.1–94.0)	48.2
		CRC	55	87.3 (74.9–94.3)	68.8
		Control	65	18.5 (10.3–30.4)	N/A
3/3 and mean Ct <39	2/3	AA	6	83.3 (36.5–99.1)	71.0
		CRC	55	89.1 (77.1–95.5)	76.8
		Control	65	12.3 (5.8–23.4)	N/A
3/3 and mean Ct <39	3/3	AA	6	66.7 (24.1–94.0)	59.0
		CRC	55	87.3 (74.9–94.3)	79.6
		Control	65	7.7 (2.9–17.8)	N/A
3/3 and mean Ct <38	2/3	AA	6	66.7 (24.1–94.0)	57.4
		CRC	55	89.1 (77.1–95.5)	79.9
		Control	65	9.2 (3.8–19.7)	N/A
3/3 and mean Ct <38	3/3	AA	6	50.0 (13.9–86.1)	43.8
		CRC	55	87.3 (74.9–94.3)	81.1
		Control	65	6.2 (2.0–15.8)	N/A

**N/A, not applicable.**

**FIGURE 2:**
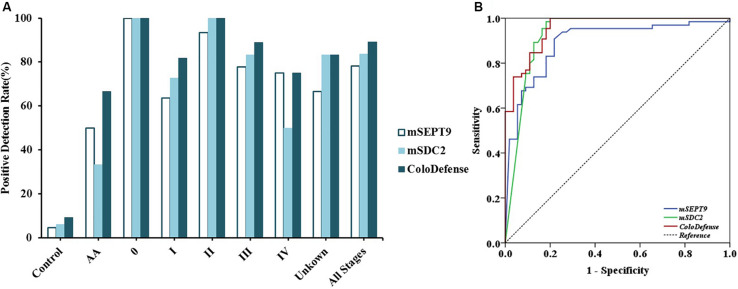
Performance of ColoDefense test in detecting control, AA and CRC across stages 0–IV in the training set. **(A)** Positive detection rates for control and all stages of CRC. **(B)** ROC curves for ColoDefense test in detecting CRC.

Out of 126 stool samples in the training set, m*SEPT9* was detected in 4.6% of control, 50.0% of AA, 100.0% of stage 0 CRC (1/1), 63.6% of stage I CRC (7/11), 93.3% of stage II CRC (14/15), 77.8% of stage III CRC (14/18), 75.0% of stage IV CRC (3/4), and 66.7% of unknown stage CRC (4/6) samples (Figure 2A). m*SDC2* was detected in 6.2% of control, 33.3% of AA, 100.0% of stage 0 CRC (1/1), 72.7% of stage I CRC (8/11), 100.0% of stage II CRC (15/15), 83.3% of stage III CRC (15/18), 50.0% of stage IV CRC (2/4), and 83.3% of unknown stage CRC (5/6) samples. In contrast, with ColoDefense test, the positive detection rates improved to 66.7% (4/6) for AA, 100.0% (1/1) for stage 0 CRC, 81.8% for stage I CRC (9/11), 100.0% for stage II CRC (15/15), 88.9% for stage III CRC (16/18), 75.0% for stage IV CRC (3/4), and 83.3% of unknown stage CRC (5/6), and the positive detection rate for control was 9.2% (refer to specificity was 90.8%) (Supplementary Table 3). AUC for m*SEPT9* alone in detecting CRC was 0.892 (95% CI: 0.831–0.953), and AUC for m*SDC2* alone in detecting CRC was 0.930 (95% CI: 0.877–0.983) (Figure 2B). In contrast, ColoDefense test improved AUC to 0.956 (95% CI: 0.924–0.988).

**FIGURE 3:**
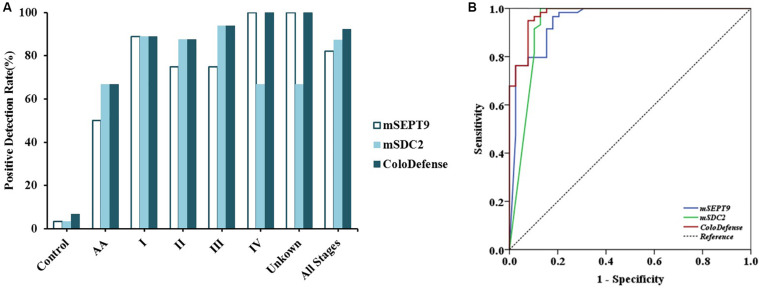
Performance of ColoDefense test in detecting control, AA and CRC across stages I–IV in the validation set. **(A)** Positive detection rates for control and all stages of CRC. **(B)** ROC curves for ColoDefense test in detecting CRC.

To validate the performance of the cut-off value of ColoDefense for stool samples, we enrolled an independent cohort of 104 subjects as a validation set. In the validation set, m*SEPT9* showed 50.0% (95% CI: 13.9–86.1%) and 82.1% (95% CI: 65.9–91.9%) sensitivities and 96.6% (95% CI: 87.3–99.4%) specificity for detecting AA and CRC, and m*SDC2* showed 66.7% (95% CI: 24.1–94.0%) and 87.2% (95% CI: 71.8–95.2%) sensitivities and 96.6% (95% CI: 87.3–99.4%) specificity for detecting AA and CRC (Figure 3A). In contrast, ColoDefense test improved the sensitivities to 66.7% (95% CI: 24.1–94.0%) for AA and 92.3% (95% CI: 78.0–98.0%) for CRC with a specificity of 93.2% (95% CI: 82.7–97.8%) (Supplementary Table 3). AUC for m*SEPT9* alone in detecting CRC was 0.948 (95% CI: 0.901–0.995), and AUC for m*SDC2* alone in detecting CRC was 0.937 (95% CI: 0.875–0.999), whereas ColoDefense test improved the AUC to 0.977 (95% CI: 0.952–1.000) (Figure 3B).

Furthermore, there was no significant difference for the positive detection rates of m*SEPT9* alone, m*SDC2* alone or ColoDefense test between different stage, age groups or genders (*p* > 0.05, Table 4). The positive detection rates of m*SEPT9* showed significant difference among different tumor locations (*p* < 0.05), and the positive detection rates of mS*DC2* seemed to increase with the increase of tumor sizes (*p* < 0.05). However, when m*SEPT9* with mS*DC2* were combined as ColoDefense test, no significant difference was apparent in detecting CRC among different locations or tumor sizes.

**TABLE 4:** Results of ColoDefense test in detecting CRC for different stage, age groups, genders, tumor locations, and tumor sizes.

	**m*SEPT9* positive detection rate (%)**	***p*-value**	**m*SDC2* positive detection rate (%)**	***p*-value**	**ColoDefense positive detection rate (%)**	***p*-value**
**Stage**						
I–II (*n* = 44)	81.8(36/44)	0.664	88.6(39/44)	0.450	90.9(40/44)	0.916
III–IV (*n* = 41)	78.1(32/41)		82.9(34/41)		90.2(37/41)	
N/A (*n* = 9)	77.8(7/9)		77.8(7/9)		88.9(8/9)	
**Age**						
<60 (*n* = 48)	79.2(38/48)	0.878	85.4(41/48)	0.931	93.8(45/48)	0.263
≥60 (*n* = 46)	80.4(37/46)		84.8(39/46)		87.0(40/46)	
**Gender**						
Male (*n* = 53)	86.8(46/53)	0.054	86.8(46/53)	0.602	90.6(48/53)	0.985
Female (*n* = 41)	70.7(29/41)		82.9(34/41)		90.2(37/41)	
**Location**						
Proximal (*n* = 46)	89.1(41/46)	0.031*	84.8(39/46)	0.113	93.5(43/46)	0.361
Distal (*n* = 41)	70.7(29/41)		87.8(29/41)		87.8(36/41)	
N/A (*n* = 7)	71.4(5/7)		71.4(5/7)		85.7(6/7)	
**Size**						
<3 cm (*n* = 11)	81.8(9/11)	0.870^a^	54.6(6/11)	0.003**^a^	81.8(9/11)	0.324^a^
3–6 cm (*n* = 59)	79.7(47/59)	0.719^b^	89.8(53/59)	0.026*^b^	91.5(54/59)	0.202^b^
>6 cm (*n* = 8)	75.0(6/8)	0.761^c^	100.0(8/8)	0.345^c^	100.0(8/8)	0.392^c^
N/A (*n* = 16)	81.3(13/16)		81.3(13/16)		87.5(14/16)	

*p < 0.05; **p < 0.01. N/A, not applicable. ^*a*^p-value between <3 cm and 3–6 cm. ^*b*^p-value between <3 cm and >6 cm. ^*c*^p-value between 3–6 cm and >6 cm.

## Discussion

As one of the most common cancers globally, CRC caused millions of new cases and deaths in 2018 (Bray et al., 2018). Longstanding and early screening program is the most effective way to reduce the incidence and mortality of CRC. Up to know, several strategies have been demonstrated to be effective for CRC early screening, including colonoscopy, fecal immunochemical test, and high-sensitivity gFOBT (Wolf et al., 2018; Siegel et al., 2019). In 2018, multitarget stool DNA test was recommended as a new CRC screening method in the updated guideline from American Cancer Society (ACS) for adults elder than 45 years with an average risk of CRC (Wolf et al., 2018). In this study, we optimized the cut-off value of ColoDefense test for stool samples and evaluated the feasibility of stool ColoDefense test for CRC early detection.

In our previous study, we demonstrated that ColoDefense test detected 47.8% AA, 87.1% early stage (0–II) CRC and 88.9% stage I–IV CRC, with a specificity of 92.8% for plasma samples (Zhao et al., 2019). And plasma ColoDefense test showed better performance than m*SEPT9* or m*SDC2* alone in detecting AA and CRC. In the present study, we attempted to determine whether stool ColoDefense test was a viable option for CRC early detection. The results showed that the sensitivities of stool ColoDefense test in detecting AA, early stage (0–II) CRC and stage I–IV CRC were 66.7% (8/12), 90.9% (40/44), and 90.4% (85/94), respectively, with a specificity of 91.9%. Compared with plasma ColoDefense test, stool ColoDefense test showed better performance in detection AA (66.7 vs. 47.8%) and stage I CRC (85.0 vs. 80.0%). This was likely because DNA in stool samples originates directly from the cells of the intestinal wall where precancerous and tumor tissues are located, while ctDNA in plasma has passed through various barriers within the body to reach the circulation. Therefore, the higher DNA abundance in stool samples than that in plasma resulted in higher sensitivities in AA and early stage CRC detection, which is also consistent with the different cut-off values between stool and plasma ColoDefense test. Another advantage of stool ColoDefense test is that sample can be collected at home and sent to laboratories for analysis, a convenience desirable for those people who are concerned with privacy or too busy to go to the hospital. On the contrary, plasma ColoDefense test may be the better choice for hospitals and other medical institutions. Blood draw is more convenient for medical personnel to perform, and the pretreatment of blood samples is easier than that of stool samples.

In previous studies, several stool methylated DNA biomarkers have been investigated for CRC early detection. Glöckner et al. (2009) developed a methylation-specific PCR (MSP) assay for methylated *TFPI2*, which could detect stage I to III CRC patients with a sensitivity of 76–89% and a specificity of 79–93% with stool samples. Tang et al. (2011) reported a MSP assay for methylated *SFRP2*, which could detect 84.0% CRC with a specificity of 54.0% with stool DNA. Both methylated *TFPI2* and *SFRP2* tests employed agarose gel electrophoresis-based MSP approach, not suitable for clinical application.

Oh et al. (2017) recently reported a stool m*SDC2* assay based on a nested-PCR method (LTE-qMSP) for early CRC screening, which detected 90.0% CRC and 33.3% small polyps with a specificity of 90.9%. Niu et al. (2017) also published a qPCR based m*SDC2* assay in 2017, which detected 81.1% of CRC and 58.2% of >1 cm adenomas with a specificity of 93.3%. Both results are comparable to the results of m*SDC2* alone in our study, indicating that m*SDC2* has a high potential as an early diagnostic biomarker for CRC. However, the results in our study indicated that m*SDC2* alone may result in more false negatives (Figures 2, 3) due to its different sensitivities for tumors of different sizes (Table 4). In contrast, the combination of m*SDC2* and m*SEPT9* for stool DNA test led to higher sensitivities for all CRC stages (Figure 3) and the best balance of positive detection rates for different age groups, genders, tumor locations, and tumor sizes (Table 4), which was also demonstrated for plasma ColoDefense test (Zhao et al., 2019).

Cologuard, a stool DNA test approved by FDA for CRC screening in 2014, employs multiple biomarkers including two methylated genes (*BMP3* and *NDRG4*), 7 *KRAS* mutation sites and an immunochemical assay for human hemoglobin. It detected CRC and AA with 92.3 and 42.4% sensitivities, respectively, and a specificity of 86.7% (Imperiale et al., 2014). As the first FDA approved test for CRC screening using multiple biomarkers demonstrating impressive sensitivity and specificity, Cologuard has been recommended by ACS as an option for CRC screening (Wolf et al., 2018). Compared with Cologuard, stool ColoDefense test only requires approximately 5 g stool for each subject and detects two methylation biomarkers and an internal control gene in one multiplex qPCR reaction, resulting in significantly lower cost and much simpler procedure. Moreover, stool ColoDefense test achieved a higher sensitivity for AA (66.7 vs. 42.4%) and a similar sensitivity of CRC (90.4 vs. 92.3%) with a high specificity of 91.9%. Therefore, stool ColoDefense test may be a superb alternative for CRC screening in developing countries such as China.

However, there are a few limitations in this study. For example, the numbers of AA samples, stage IV CRC samples, and the total number of clinical samples examined were relatively small, which could lead to fluctuations of estimates of performance. This can be improved by increasing the number of enrolled patients in future studies. In this study, we analyzed performance of stool samples in a training and a validation set as a prospective study, but more validation studies in multiple clinical centers as well as a large prospective study within a population screening program should be carried out in the future. In addition, as non-advanced colon polyps were prevalent in 7.4–52.5% of patients undergoing screening colonoscopy (Corley et al., 2014), the performance of ColoDefense test on non-advanced colon polyps should also be examined in further studies.

## Conclusion

Stool ColoDefense test demonstrated high sensitivities for AA and CRC detection, which were higher than those of either m*SEPT9* or m*SDC2* alone. Therefore, stool ColoDefense test has the potential to become a powerful, convenient and highly effective screening tool for CRC early detection.

## Data Availability Statement

The datasets used and/or analyzed during the current study are available from the corresponding author on reasonable request.

## Ethics Statement

The studies involving human participants were reviewed and approved by the Institutional Review Board of the Affiliated Hospital of Xuzhou Medical University (Ethics Committee reference number: XYFY2018-KL081). The patients/participants provided their written informed consent to participate in this study.

## Author Contributions

GZ, XL, and YM performed the statistical analyses and drafted the manuscript. YL, HL, SL, YZ, and JM participated in sample collection and data analysis. GZ, SX, SF, and MZ conceived of the study and participated in the design and coordination of the study. All authors read and approved the final manuscript.

## Conflict of Interest

GZ and SX were employees of Suzhou VersaBio Technologies Co., Ltd. SX was the shareholder of Suzhou VersaBio Technologies Co., Ltd. The remaining authors declare that the research was conducted in the absence of any commercial or financial relationships that could be construed as a potential conflict of interest.
